# Diaqua­bis(2,2′-biimidazole)cobalt(II) 4,4′-dicarboxy­biphenyl-3,3′-di­car­boxylate

**DOI:** 10.1107/S1600536809007764

**Published:** 2009-03-11

**Authors:** Jie Kang, Chang-Cang Huang, Lai-Sheng Zhai, Xiao-Huan Qin, Zhong-Qian Liu

**Affiliations:** a College of Pharmacy, Fujian Medical University, Fuzhou, Fujian 350004, People’s Republic of China; b State Key Laboratory Breeding Base of Photocatalysis, Fuzhou University, Fuzhou 350002, People’s Republic of China

## Abstract

In the title compound, [Co(C_6_H_6_N_4_)_2_(H_2_O)_2_](C_16_H_8_O_8_), the Co^II^ cation and the organic anion occupy different crystallographic inversion centres and, as a consequence, the asymmetric unit comprises two half-mol­ecules. The benzene groups are coplanar. The four coordinating N atoms of the two bidentate biimidazole ligands define the equatorial plane of a slightly distorted octa­hedral CoO_2_N_4_ geometry, and the water O atoms lie in the axial coordination sites. Translational (*a*,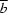) and inversion-related symmetry operations link the Co complex mol­ecules and the negatively charged carboxyl­ate anions *via* inter­molecular N—H⋯O and O—H⋯O hydrogen bonds into sheets parallel to (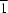
01). The coordinated water mol­ecules connect the sheets through O—H⋯O hydrogen bonds, forming a three-dimensional framework. In addition, two intra­molecular O—H⋯O hydrogen bonds are observed between the carboxyl and carboxyl­ate groups.

## Related literature

For a review on organic–inorganic hybrid materials, see: Hagrman *et al.* (1999 ▶). For a tetra­nuclear cobalt complex with a 1,2,4-benzene­tricarboxyl­ate linker, see: Jia *et al.* (2007 ▶). For a highly porous metal-organic framework with a benzene­dicarboxyl­ate linker, see: Li *et al.* (1999 ▶). For coordination polymers of Ag(I), Cd(II) and Zn(II) with the flexible 2-(1*H*-imidazole-1-yl)acetic acid linker, see: Wang *et al.* (2007 ▶). For the structure of 1,1′-biphenyl-2,3,3′,4′-tetra­carboxylic acid monohydrate and related structures cited therein, see: Jiang *et al.* (2008 ▶).
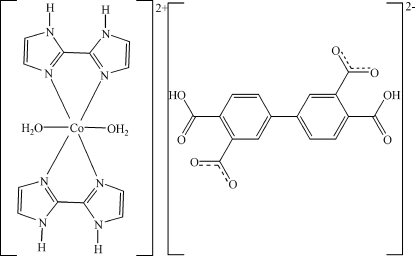


## Experimental

### 

#### Crystal data

[Co(C_6_H_6_N_4_)_2_(H_2_O)_2_](C_16_H_8_O_8_)
*M* *_r_* = 691.48
Triclinic, 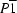

*a* = 8.2272 (16) Å
*b* = 9.772 (2) Å
*c* = 10.484 (2) Å
α = 63.81 (3)°
β = 67.93 (3)°
γ = 84.03 (3)°
*V* = 699.0 (2) Å^3^
*Z* = 1
Mo *K*α radiation
μ = 0.69 mm^−1^
*T* = 293 K
0.42 × 0.26 × 0.20 mm

#### Data collection

Bruker APEXII CCD area-detector diffractometer
Absorption correction: multi-scan (*SADABS*; Bruker, 2001 ▶) *T*_min_ = 0.760, *T*_max_ = 0.874
4982 measured reflections
2603 independent reflections
2527 reflections with *I* > 2σ(*I*)
*R*_int_ = 0.022

#### Refinement

*R*[*F* ^2^ > 2σ(*F* ^2^)] = 0.033
*wR*(*F* ^2^) = 0.087
*S* = 1.01
2603 reflections
227 parameters
5 restraints
H atoms treated by a mixture of independent and constrained refinement
Δρ_max_ = 0.31 e Å^−3^
Δρ_min_ = −0.21 e Å^−3^

### 

Data collection: *APEX2* (Bruker, 2004 ▶); cell refinement: *SAINT-Plus* (Bruker, 2001 ▶); data reduction: *SAINT-Plus*; program(s) used to solve structure: *SHELXS97* (Sheldrick, 2008 ▶); program(s) used to refine structure: *SHELXL97* (Sheldrick, 2008 ▶); molecular graphics: *SHELXTL* (Sheldrick, 2008 ▶); software used to prepare material for publication: *SHELXTL*.

## Supplementary Material

Crystal structure: contains datablocks global, I. DOI: 10.1107/S1600536809007764/si2153sup1.cif

Structure factors: contains datablocks I. DOI: 10.1107/S1600536809007764/si2153Isup2.hkl

Additional supplementary materials:  crystallographic information; 3D view; checkCIF report

## Figures and Tables

**Table:** 

Co1—O5	2.0882 (19)
Co1—N1	2.1412 (16)
Co1—N3	2.1579 (16)

**Table:** 

O5—Co1—N1	88.32 (7)
N1—Co1—N3^i^	100.77 (6)
O5—Co1—N3	87.82 (7)

Symmetry code: (i) 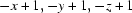.

**Table 2:** Hydrogen-bond geometry (Å, °)

*D*—H⋯*A*	*D*—H	H⋯*A*	*D*⋯*A*	*D*—H⋯*A*
N2—H2*A*⋯O1^ii^	0.92 (2)	1.87 (2)	2.791 (3)	178.8 (18)
N4—H4*A*⋯O2^ii^	0.920 (18)	1.897 (19)	2.808 (3)	170.3 (19)
O5—H1*W*⋯O1^iii^	0.82 (2)	1.93 (2)	2.739 (3)	169 (2)
O5—H2*W*⋯O4^i^	0.81 (2)	1.88 (2)	2.673 (3)	163 (2)
O3—H3⋯O2	0.82	1.62	2.432 (3)	172

Symmetry codes: (i) 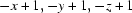; (ii) 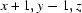; (iii) 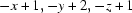.

**Fig. 1.**
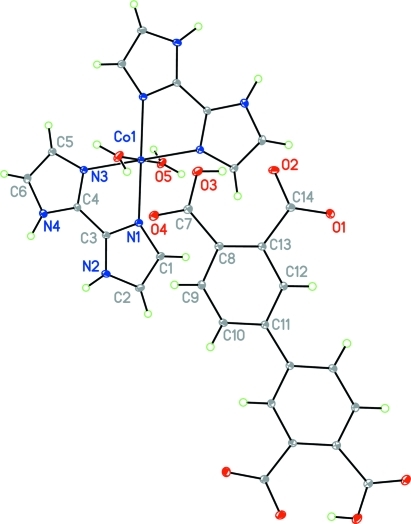
The molecular structure of the title compound with displacement ellipsoids drawn at the 10% probability level. Symmetry-related atoms are unlabelled. Symmetry code for the Co-complex: (1 - x, 1 - y, 1 - z); symmetry code for the organic anion: (1 - x, 2 - y, 2 - z).

**Fig. 2.**
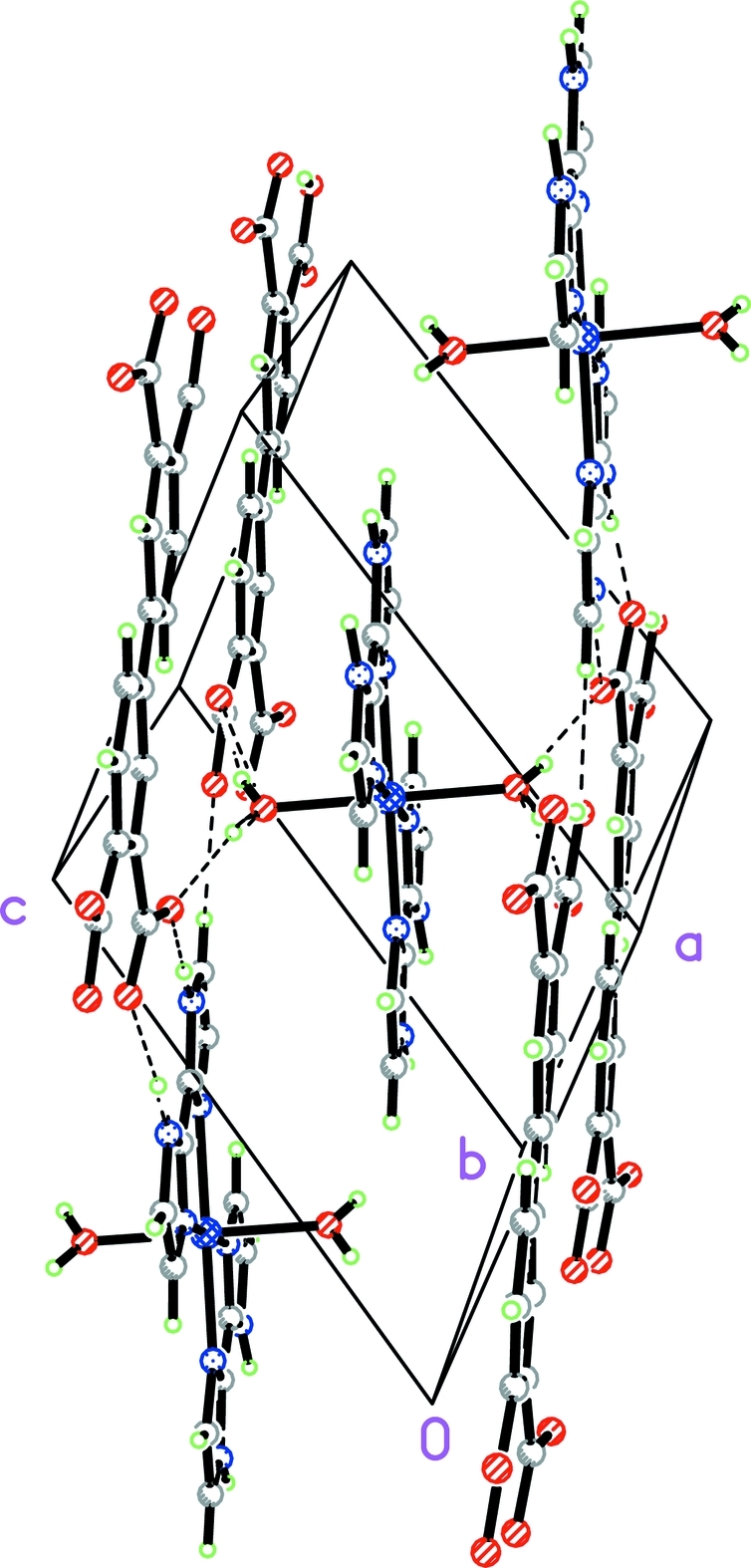
A packing view of the title compound. Hydrogen bonds are marked by dashed lines.

## supplementary crystallographic information

### Comment

Design and construction of metal-organic frameworks (MOFs) have attracted considerable attention in recent years, not only for their intriguing structural motifs (Wang *et al.* 2007), but also for their potential applications, e. g. as organic-inorganic hybrid materials (Hagrman *et al.*,  1999), highly porous metal-organic framework (Li *et al.*,  1999), magnetochemistry (Jia *et al.*,  2007). In contrast, the two ionic components of the title structure interact with N—H···O and O—H···O hydrogen bonds to form a three-dimensional framework.

As shown in Fig. 1, the Co atom (site symmetry 1) is bonded to two aqua and two bidentate biimidizole ligands, to result in a slightly distorterd octahedral CoO_2_N_4_ geometry for the central metal. The Co^II^ cation and the organic anion occupy different crystallographic inversion centres, and as a consequence the asymmetric unit of the cell comprises two half molecules (Z' = 1/2), and the benzene groups are co-planar. The four nitrogen atoms belonging to two biimidizole ligands lie in the equatorial plane and the two aqua oxygen atoms lie in the axial coordination sites. Selected bond lengths and angles around Co are listed in Table 1. The 1,1'-biphenyl-3,3'-dicarboxylate-4,4'-dicarboxylic acid acts as a negative electron balance. With three kinds of hydrogen bonds (Table 2) of N2—H2A···O1, N4—H4A···O2, and O5—H1W···O1, two-dimensional planes are formed. Furthermore, a three-dimensional framework (Fig. 2) is generated with the intermolecular hydrogen bonding contact O5—H2W···O4 along the [-1 0 1] direction. The organic anion has two intramolecular O3—H3···O2 hydrogen bonds between the carboxylic acid units and the carboxylate acceptors (Table 2). In contrast to the co-planar biphenyl group of the title compound, a dihedral angle of 42.30 (11)° between the two benzene rings was observed in the structure of 1,1'-biphenyl-2,3,3',4'-tetracarboxylic acid monohydrate (Jiang *et al.*  2008).

### Experimental

All chemicals and Teflon-lined stainless steel autoclave were purchased from Jinan Henghua Sci. & Tec. Co. Ltd. A mixture of 3,3',4,4'-biphenyl tetracarboxylic acid (0.1 mmoL), cobalt(II) acetate (0.1 mmoL), and diimdazole (0.1 mmoL) in 10 ml distilled water sealed in a 25 ml Teflon-lined stainless steel autoclave was kept at 433 K for three days. Yellow crystals suitable for the X-ray experiment were obtained.

### Refinement

The H atoms of the water molecule were located from difference density maps and were refined with distance restraints of d(H–H) = 1.38 (2) Å, d(O–H) = 0.88 (2) Å, and with a fixed *U*_iso_ of 0.056 Å^2^. All other H atoms were placed in calculated positions with a C—H bond distance of 0.93 Å and *U*_iso_(H) = 1.2*U*_eq_ of the carrier atom.

### Crystal data

**Table:**

[Co(C_6_H_6_N_4_)_2_(H_2_O)_2_](C_16_H_8_O_8_)	*Z* = 1
*M**_r_* = 691.48	*F*(000) = 355
Triclinic, *P*1	*D*_x_ = 1.643 Mg m^−^^3^
Hall symbol: -P 1	Mo *K*α radiation, λ = 0.71073 Å
*a* = 8.2272 (16) Å	Cell parameters from 2603 reflections
*b* = 9.772 (2) Å	θ = 3.1–25.8°
*c* = 10.484 (2) Å	µ = 0.69 mm^−^^1^
α = 63.81 (3)°	*T* = 293 K
β = 67.93 (3)°	Block, colorless
γ = 84.03 (3)°	0.42 × 0.26 × 0.20 mm
*V* = 699.0 (2) Å^3^	

### Data collection

**Table:**

Bruker APEXII CCD area-detector diffractometer	2603 independent reflections
Radiation source: fine-focus sealed tube	2527 reflections with *I* > 2σ(*I*)
graphite	*R*_int_ = 0.022
φ and ω scans	θ_max_ = 25.8°, θ_min_ = 3.1°
Absorption correction: multi-scan (*SADABS*; Bruker, 2001)	*h* = −10→9
*T*_min_ = 0.760, *T*_max_ = 0.874	*k* = −11→11
4982 measured reflections	*l* = −12→12

### Refinement

**Table:**

Refinement on *F*^2^	Primary atom site location: structure-invariant direct methods
Least-squares matrix: full	Secondary atom site location: difference Fourier map
*R*[*F*^2^ > 2σ(*F*^2^)] = 0.033	Hydrogen site location: inferred from neighbouring sites
*wR*(*F*^2^) = 0.087	H atoms treated by a mixture of independent and constrained refinement
*S* = 1.01	*w* = 1/[σ^2^(*F*_o_^2^) + (0.045*P*)^2^ + 0.376*P*] where *P* = (*F*_o_^2^ + 2*F*_c_^2^)/3
2603 reflections	(Δ/σ)_max_ = 0.001
227 parameters	Δρ_max_ = 0.31 e Å^−^^3^
5 restraints	Δρ_min_ = −0.21 e Å^−^^3^

### Special details

**Table:**

Geometry. All e.s.d.'s (except the e.s.d. in the dihedral angle between two l.s. planes) are estimated using the full covariance matrix. The cell e.s.d.'s are taken into account individually in the estimation of e.s.d.'s in distances, angles and torsion angles; correlations between e.s.d.'s in cell parameters are only used when they are defined by crystal symmetry. An approximate (isotropic) treatment of cell e.s.d.'s is used for estimating e.s.d.'s involving l.s. planes.
Refinement. Refinement of *F*^2^ against ALL reflections. The weighted *R*-factor *wR* and goodness of fit *S* are based on *F*^2^, conventional *R*-factors *R* are based on *F*, with *F* set to zero for negative *F*^2^. The threshold expression of *F*^2^ > σ(*F*^2^) is used only for calculating *R*-factors(gt) *etc*. and is not relevant to the choice of reflections for refinement. *R*-factors based on *F*^2^ are statistically about twice as large as those based on *F*, and *R*- factors based on ALL data will be even larger.

### Fractional atomic coordinates and isotropic or equivalent isotropic displacement parameters (Å^2^)

**Table:**

	*x*	*y*	*z*	*U*_iso_*/*U*_eq_	
Co1	0.5000	0.5000	0.5000	0.03059 (13)	
C1	0.7400 (3)	0.5910 (2)	0.6513 (3)	0.0388 (5)	
H1	0.7036	0.6865	0.6446	0.047*	
C2	0.8671 (3)	0.5187 (2)	0.7049 (3)	0.0403 (5)	
H2	0.9323	0.5542	0.7415	0.048*	
C3	0.7617 (2)	0.3779 (2)	0.6368 (2)	0.0303 (4)	
C4	0.7231 (2)	0.2591 (2)	0.6037 (2)	0.0305 (4)	
C5	0.5986 (3)	0.1500 (2)	0.5229 (3)	0.0396 (5)	
H5	0.5266	0.1311	0.4812	0.047*	
C6	0.7143 (3)	0.0532 (2)	0.5751 (3)	0.0428 (5)	
H6	0.7355	−0.0425	0.5762	0.051*	
C7	0.2215 (3)	0.6649 (2)	0.8670 (2)	0.0355 (4)	
C8	0.2957 (2)	0.7806 (2)	0.8950 (2)	0.0300 (4)	
C9	0.4327 (3)	0.7276 (2)	0.9485 (2)	0.0372 (5)	
H9	0.4710	0.6319	0.9577	0.045*	
C10	0.5135 (3)	0.8106 (2)	0.9882 (3)	0.0383 (5)	
H10	0.6051	0.7708	1.0222	0.046*	
C11	0.4595 (2)	0.9535 (2)	0.9779 (2)	0.0289 (4)	
C12	0.3260 (2)	1.0084 (2)	0.9211 (2)	0.0301 (4)	
H12	0.2894	1.1046	0.9116	0.036*	
C13	0.2436 (2)	0.9278 (2)	0.8777 (2)	0.0279 (4)	
C14	0.1084 (2)	1.0170 (2)	0.8107 (2)	0.0330 (4)	
N1	0.6735 (2)	0.50249 (18)	0.60857 (19)	0.0340 (4)	
N2	0.8799 (2)	0.3839 (2)	0.6943 (2)	0.0365 (4)	
H2A	0.951 (3)	0.307 (2)	0.726 (2)	0.044*	
N3	0.6043 (2)	0.27901 (17)	0.54115 (19)	0.0335 (4)	
N4	0.7934 (2)	0.12422 (19)	0.6258 (2)	0.0371 (4)	
H4A	0.869 (2)	0.078 (2)	0.676 (2)	0.045*	
O1	0.0983 (2)	1.15279 (16)	0.7860 (2)	0.0492 (4)	
O2	0.0097 (2)	0.95144 (19)	0.7838 (2)	0.0552 (5)	
O3	0.0823 (2)	0.68970 (19)	0.8329 (2)	0.0546 (4)	
H3	0.0513	0.7749	0.8241	0.082*	
O4	0.2931 (2)	0.54625 (18)	0.8800 (2)	0.0500 (4)	
O5	0.7116 (2)	0.58986 (18)	0.29083 (18)	0.0469 (4)	
H1W	0.763 (3)	0.6721 (18)	0.259 (3)	0.056*	
H2W	0.730 (3)	0.557 (2)	0.228 (2)	0.056*	

### Atomic displacement parameters (Å^2^)

**Table:**

	*U*^11^	*U*^22^	*U*^33^	*U*^12^	*U*^13^	*U*^23^
Co1	0.0353 (2)	0.0244 (2)	0.0457 (2)	0.00797 (14)	−0.02832 (17)	−0.01710 (17)
C1	0.0454 (11)	0.0331 (11)	0.0579 (13)	0.0098 (9)	−0.0324 (10)	−0.0271 (10)
C2	0.0445 (11)	0.0408 (12)	0.0564 (13)	0.0074 (9)	−0.0326 (10)	−0.0281 (10)
C3	0.0306 (9)	0.0293 (10)	0.0390 (10)	0.0070 (7)	−0.0211 (8)	−0.0158 (8)
C4	0.0343 (10)	0.0251 (9)	0.0380 (10)	0.0072 (7)	−0.0206 (8)	−0.0140 (8)
C5	0.0514 (12)	0.0301 (10)	0.0528 (12)	0.0054 (9)	−0.0327 (10)	−0.0206 (9)
C6	0.0559 (13)	0.0272 (10)	0.0599 (13)	0.0106 (9)	−0.0321 (11)	−0.0240 (10)
C7	0.0417 (11)	0.0327 (11)	0.0414 (11)	0.0038 (8)	−0.0214 (9)	−0.0194 (9)
C8	0.0341 (9)	0.0292 (10)	0.0327 (9)	0.0033 (7)	−0.0170 (8)	−0.0150 (8)
C9	0.0479 (11)	0.0277 (10)	0.0519 (12)	0.0141 (8)	−0.0317 (10)	−0.0222 (9)
C10	0.0468 (11)	0.0320 (10)	0.0561 (12)	0.0159 (9)	−0.0388 (10)	−0.0227 (9)
C11	0.0341 (9)	0.0250 (9)	0.0348 (9)	0.0058 (7)	−0.0202 (8)	−0.0139 (8)
C12	0.0337 (9)	0.0254 (9)	0.0389 (10)	0.0071 (7)	−0.0210 (8)	−0.0153 (8)
C13	0.0286 (9)	0.0276 (9)	0.0326 (9)	0.0043 (7)	−0.0176 (7)	−0.0126 (8)
C14	0.0321 (10)	0.0332 (11)	0.0443 (11)	0.0072 (8)	−0.0229 (8)	−0.0197 (9)
N1	0.0382 (9)	0.0291 (9)	0.0491 (10)	0.0093 (7)	−0.0287 (8)	−0.0205 (8)
N2	0.0371 (9)	0.0364 (9)	0.0505 (10)	0.0116 (7)	−0.0303 (8)	−0.0215 (8)
N3	0.0399 (9)	0.0260 (8)	0.0451 (9)	0.0066 (7)	−0.0273 (8)	−0.0159 (7)
N4	0.0416 (9)	0.0299 (9)	0.0503 (10)	0.0115 (7)	−0.0286 (8)	−0.0187 (8)
O1	0.0495 (9)	0.0308 (8)	0.0865 (12)	0.0132 (7)	−0.0501 (9)	−0.0234 (8)
O2	0.0614 (10)	0.0485 (9)	0.0989 (13)	0.0239 (8)	−0.0639 (10)	−0.0445 (9)
O3	0.0583 (10)	0.0429 (9)	0.0953 (13)	0.0131 (7)	−0.0519 (10)	−0.0402 (10)
O4	0.0654 (10)	0.0392 (9)	0.0746 (11)	0.0165 (7)	−0.0444 (9)	−0.0368 (8)
O5	0.0579 (10)	0.0382 (9)	0.0516 (9)	−0.0085 (7)	−0.0184 (8)	−0.0244 (7)

### Geometric parameters (Å, °)

**Table:**

Co1—O5^i^	2.0882 (19)	C7—O4	1.220 (3)
Co1—O5	2.0882 (19)	C7—O3	1.293 (2)
Co1—N1	2.1412 (16)	C7—C8	1.519 (3)
Co1—N1^i^	2.1412 (16)	C8—C9	1.397 (3)
Co1—N3^i^	2.1579 (16)	C8—C13	1.410 (3)
Co1—N3	2.1579 (16)	C9—C10	1.375 (3)
C1—C2	1.359 (3)	C9—H9	0.9300
C1—N1	1.369 (2)	C10—C11	1.390 (3)
C1—H1	0.9300	C10—H10	0.9300
C2—N2	1.360 (3)	C11—C12	1.391 (3)
C2—H2	0.9300	C11—C11^ii^	1.490 (3)
C3—N1	1.325 (2)	C12—C13	1.395 (3)
C3—N2	1.340 (2)	C12—H12	0.9300
C3—C4	1.444 (3)	C13—C14	1.528 (3)
C4—N3	1.324 (2)	C14—O1	1.235 (2)
C4—N4	1.341 (2)	C14—O2	1.261 (2)
C5—C6	1.360 (3)	N2—H2A	0.921 (10)
C5—N3	1.364 (2)	N4—H4A	0.918 (10)
C5—H5	0.9300	O3—H3	0.8200
C6—N4	1.366 (3)	O5—H1W	0.82 (2)
C6—H6	0.9300	O5—H2W	0.81 (2)
			
O5^i^—Co1—O5	180	C13—C8—C7	129.44 (17)
O5^i^—Co1—N1	91.68 (7)	C10—C9—C8	122.91 (18)
O5—Co1—N1	88.32 (7)	C10—C9—H9	118.5
O5^i^—Co1—N1^i^	88.32 (7)	C8—C9—H9	118.5
N1—Co1—N1^i^	180	C9—C10—C11	120.56 (18)
O5^i^—Co1—N3^i^	87.82 (7)	C9—C10—H10	119.7
O5—Co1—N3^i^	92.18 (7)	C11—C10—H10	119.7
N1—Co1—N3^i^	100.77 (6)	C10—C11—C12	116.73 (17)
N1^i^—Co1—N3^i^	79.23 (6)	C10—C11—C11^ii^	122.6 (2)
O5^i^—Co1—N3	92.18 (7)	C12—C11—C11^ii^	120.7 (2)
O5—Co1—N3	87.82 (7)	C11—C12—C13	123.93 (17)
N1—Co1—N3	79.23 (6)	C11—C12—H12	118.0
N3^i^—Co1—N3	180	C13—C12—H12	118.0
C2—C1—N1	109.90 (17)	C12—C13—C8	118.32 (16)
C2—C1—H1	125.0	C12—C13—C14	113.67 (16)
N1—C1—H1	125.0	C8—C13—C14	127.98 (16)
N2—C2—C1	106.11 (17)	O1—C14—O2	121.81 (18)
N2—C2—H2	126.9	O1—C14—C13	118.10 (17)
C1—C2—H2	126.9	O2—C14—C13	120.09 (17)
N1—C3—N2	111.51 (17)	C3—N1—C1	105.05 (16)
N1—C3—C4	119.29 (16)	C3—N1—Co1	111.21 (12)
N2—C3—C4	129.20 (17)	C1—N1—Co1	143.48 (13)
N3—C4—N4	111.61 (17)	C3—N2—C2	107.42 (17)
N3—C4—C3	119.34 (16)	C3—N2—H2A	125.3 (15)
N4—C4—C3	129.05 (17)	C2—N2—H2A	127.2 (15)
C6—C5—N3	109.48 (18)	C4—N3—C5	105.57 (16)
C6—C5—H5	125.3	C4—N3—Co1	110.75 (12)
N3—C5—H5	125.3	C5—N3—Co1	143.64 (14)
C5—C6—N4	106.57 (17)	C4—N4—C6	106.77 (17)
C5—C6—H6	126.7	C4—N4—H4A	129.4 (15)
N4—C6—H6	126.7	C6—N4—H4A	123.4 (15)
O4—C7—O3	120.13 (18)	C7—O3—H3	109.5
O4—C7—C8	119.10 (18)	Co1—O5—H1W	121.0 (16)
O3—C7—C8	120.75 (17)	Co1—O5—H2W	121.0 (16)
C9—C8—C13	117.47 (17)	H1W—O5—H2W	116.1 (17)
C9—C8—C7	113.08 (16)		

Symmetry codes: (i) −*x*+1, −*y*+1, −*z*+1; (ii) −*x*+1, −*y*+2, −*z*+2.

### Hydrogen-bond geometry (Å, °)

**Table:**

*D*—H···*A*	*D*—H	H···*A*	*D*···*A*	*D*—H···*A*
N2—H2A···O1^iii^	0.92 (2)	1.87 (2)	2.791 (3)	179 (2)
N4—H4A···O2^iii^	0.92 (2)	1.90 (2)	2.808 (3)	170 (2)
O5—H1W···O1^iv^	0.82 (2)	1.93 (2)	2.739 (3)	169 (2)
O5—H2W···O4^i^	0.81 (2)	1.88 (2)	2.673 (3)	163 (2)
O3—H3···O2	0.82	1.62	2.432 (3)	172

Symmetry codes: (iii) *x*+1, *y*−1, *z*; (iv) −*x*+1, −*y*+2, −*z*+1; (i) −*x*+1, −*y*+1, −*z*+1.
